# Prevalence and Correlates of Family Cancer History Knowledge and Communication Among US Adults

**DOI:** 10.5888/pcd17.200257

**Published:** 2020-11-19

**Authors:** Melinda Krakow, Camella J. Rising, Neha Trivedi, Dahye C. Yoon, Robin C. Vanderpool

**Affiliations:** 1 John D. Bower School of Population Health, University of Mississippi Medical Center, Jackson, Mississippi; 2 Health Communication and Informatics Research Branch, National Cancer Institute, Bethesda, Maryland; 3 Georgetown University, Washington, DC

## Abstract

**Introduction:**

Knowing one’s family cancer history (FCH) plays an important role in cancer prevention. Communicating health histories with relatives can increase awareness about familial cancer risk and aid health care providers in personalizing cancer prevention recommendations.

**Methods:**

This study used data from the National Cancer Institute’s 2018 Health Information National Trends Survey. We calculated frequencies and weighted population estimates for key FCH communication variables. Multivariable logistic regression models estimated associations between sociodemographic characteristics and FCH communication.

**Results:**

Findings provide the first nationally representative estimates of FCH communication. Less than one-third (31.1%) of the population reported knowing FCH very well, 70.0% had discussed FCH with at least 1 biological relative, 39.0% had discussed FCH with a health care provider, and 22.2% reported being completely confident in completing FCH on medical forms. Findings also identified key demographic factors, including sex, household income, education level, and race and ethnicity, associated with these FCH measures among the US adult population.

**Conclusion:**

Results can be used to target and tailor FCH communication interventions for patients, families, and providers.

**Summary**

**What is already known on this topic?**

Knowing one’s family cancer history (FCH) plays an important role in cancer prevention. Communicating health histories with relatives can increase awareness about familial cancer risk, and aid health care providers in personalizing cancer prevention recommendations.

**What is added by this report?**

This study provides the first nationally representative estimates of FCH knowledge, communication, and confidence completing FCH on medical forms. Findings also identify key demographic factors associated with these FCH measures in the US adult population.

**What are the implications for public health practice?**

Study findings can be used to target and tailor FCH communication interventions for patient populations, families, and providers.

## Introduction

A person’s family health history encompasses a complex set of shared genetic, behavioral, and environmental risk factors that can influence health among biological relatives. Knowing one’s family health history can help identify the risk of hereditary diseases such as cancer, where 5% to 10% of cases are inherited (1,2). For example, people with a family history of colorectal, breast, ovarian, or uterine cancers may be at higher risk for these cancers than people without a family history and should speak to a physician about this history (2,3).

A documented family cancer history (FCH) is a record of cancer diagnoses among family members that can be used to trace patterns of disease and identify family members who may benefit from changes in lifestyle, genetic counseling, and earlier or more frequent routine cancer screening (4). For example, the US Preventive Services Task Force recommends women with a first-degree relative with breast cancer begin screening in their forties, rather than age 50, the age recommended for women without this history (5). However, to benefit from screening recommendations, people must know their FCH and be able to share this information with health care providers (6). Some families do not communicate about or know their FCH despite the potential benefits of sharing this information (7–14).

To date, no nationally representative estimates exist of the prevalence of FCH communication or associated demographic factors among the US adult population. To address this gap, we analyzed data from the 2018 National Cancer Institute (NCI) Health Information National Trends Survey (HINTS) (https://hints.cancer.gov). Our first study aim was to describe the prevalence of reported FCH knowledge, FCH communication with biological relatives, FCH communication with a health care provider, and confidence completing FCH on medical forms among the US adult population. Our second study aim was to identify demographic correlates of FCH knowledge, FCH communication with biological relatives, FCH communication with a health care provider, and confidence completing FCH on medical forms. Understanding these correlates may inform approaches to targeting and tailoring interventions to help patients, families, providers, and health care systems foster FCH communication to enable personalized cancer preventive care.

## Methods

We analyzed data from NCI HINTS 5, Cycle 2 (N = 3,504); data for this survey were collected from January through May 2018 using a sampling frame of all nonvacant residential addresses. HINTS is a nationally representative, population-based survey of civilian, noninstitutionalized adults in US households. The paper-and-pencil survey is administered annually via mail to collect information on health communication and health behaviors of the general public. Administration of the HINTS survey was approved by the Westat Institutional Review Board and deemed exempt from review by the National Institutes of Health Office of Human Subjects Research. HINTS survey instruments, data sets, and detailed survey methodology reports are available at hints.cancer.gov.

HINTS collected data on the following demographic characteristics: sex (male or female), age (18–34, 35–49, 50–64, 65–74, ≥75 y), marital status (married/partnered or not married/partnered), annual household income (0–$19,999, $20,000–$34,999, $35,000–$49,999, $50,000–$74,999, ≥$75,000), education (<high school graduate, high school graduate, technical or vocational school or some college, ≥college graduate), race and ethnic identity (non-Hispanic White, Non-Hispanic Black, Hispanic, or “other” [American Indian, Native Alaskan, Asian Indian, Chinese, Filipino, Japanese, Korean, Vietnamese, Other Asian, Native Hawaiian, Guamanian or Chamorro, Samoan, and Other Pacific Islander]), geographical area classified by 2013 US Rural-Urban Continuum codes (urban or rural), and personal history of cancer (yes or no). The survey also included several items relevant to FCH communication. The HINTS survey items used to derive these measures define “family” as “first- and second-degree biological relatives; that is, your grandparents, parents, brothers and sisters, children, aunts and uncles, nieces and nephews.”

FCH knowledge was measured by asking, “A family cancer history is a record of the cancers in your family. This includes knowing if you have no history of cancers in your family. How well do you know your family’s cancer history?” Five response options ranged from “very well” to “not at all.” For analyses, we dichotomized these responses into a high level of FCH knowledge (very well/well) and a low level FCH knowledge (somewhat/a little/not at all).

FCH communication was measured by asking, “Have you ever had a discussion about your family cancer history with any of the following people? If there is no cancer in your family, and you have discussed this, please include that.” Response options included a checkbox to indicate having had a discussion with each of the following people: biological mother, biological father, biological sister(s), biological brother(s), biological children, other biological family members, or a health care provider. The option, “I have not had discussions with any of these people” was also provided. We used data from this measure to generate 2 dichotomous variables, one to indicate that FCH was discussed with at least 1 biological relative on the list (yes/no) and another to indicate that FCH was discussed with a health care provider (yes/no).

Confidence completing FCH on medical forms was assessed by the item, “How confident are you that you could complete a summary of your family cancer history on a medical form?” Five response options ranged from “completely confident” to “not confident at all.” These responses were dichotomized into high level of confidence (completely confident/very confident) and low level of confidence (somewhat/a little/not at all confident).

### Data analysis

We calculated frequencies and weighted population estimates for demographic variables and FCH measures. We constructed multivariable logistic regression models to estimate associations between demographic characteristics and each of the following dependent variables: FCH knowledge, FCH communication with at least 1 biological relative, FCH communication with a health care provider, and confidence completing FCH on medical forms. In all analyses, we recoded responses listed as “response not ascertained” or “selected in error” in the original data set as missing. We used listwise deletion to remove cases with missing data for analytic variables from the corresponding analysis. We conducted all analyses in SAS version 9.4 (SAS Institute Inc) using survey weighting procedures with a set of 50 jackknife replicate weights to account for the complex survey design in generating variance estimates, as detailed in the HINTS methodology report (15). Poststratification weightings adjusted for variations in the HINTS study sample by adjusting weighted totals to approximate known population characteristics.

## Results

The HINTS survey had a response rate of 32.8%, resulting in a final sample of 3,504 people. We found a higher response rate among women (51.0%), non-Hispanic White people (64.8%), people who live in urban areas (86.3%), and people with high socioeconomic status (vocational, technical, or some college education, 39.9%; college graduate, 28.8%; and annual household income >$75,000, 39.2%) (Table 1). Just over half (52.5%) of the population reported being married or partnered. Most (90.6%) did not report a personal history of cancer. 

**Table 1:** Weighted Population Estimates (N *=* 3,504) for Demographic Characteristics, Health Information National Trends Survey (HINTS) 5, Cycle 2, 2018

Characteristic	No.^a^ (Weighted %)
**Sex**	
Male	1,310 (49.0)
Female	1,913 (51.0)
**Age, y**	
18–34	406 (23.6)
35–49	658 (26.7)
50–64	1,113 (30.4)
65–74	736 (11.3)
≥75	504 (8.1)
**Marital status**	
Married or partnered	1,747 (52.5)
Not married or partnered	1,702 (47.5)
**Annual household income, $**	
0–19,999	579 (17.6)
20,000–34,999	428 (11.8)
35,000–49,999	404 (13.5)
50,000–74,999	567 (17.8)
≥75,000	1,109 (39.2)
**Education**	
<High school graduate	275 (9.0)
High school graduate	631 (22.3)
Vocational, technical, or some college	1,039 (39.9)
≥College graduate	1,508 (28.8)
**Race/ethnicity**	
Non-Hispanic White	1,983 (64.8)
Non-Hispanic Black	444 (10.8)
Hispanic	461 (16.0)
Other^b^	263 (8.4)
**Metropolitan area**	
Rural	489 (13.7)
Urban	3,015 (86.3)
**Personal cancer history**	
Yes	593 (9.4)
No	2,898 (90.6)

a Missing data for variables (as reported in the HINTS public codebook) included sex (n = 281), age (n = 273), marital status (n = 55), annual household income (n = 417), education (n = 51), race and ethnicity (n = 353), and personal cancer history (n = 13). Thus, categories may not add to 3,504.

b “Other” is a category consisting of American Indian, Native Alaskan, Asian Indian, Chinese, Filipino, Japanese, Korean, Vietnamese, Other Asian, Native Hawaiian, Guamanian or Chamorro, Samoan, and Other Pacific Islander self-reported race.

More than half (60.1%) of the population indicated knowing their FCH well or very well (Table 2). About one-quarter (23.9%) reported knowing their FCH “somewhat”; 16.0% indicated knowing their FCH a little or not at all.

**Table 2:** Weighted Population Estimates (N = 3,504) for Family Cancer History (FCH) Knowledge, FCH Communication, and Confidence Completing FCH on Medical Forms, Health Information National Trends Survey (HINTS) 5, Cycle 2, 2018^a^

Characteristic	No. (Weighted %)
**FCH knowledge**	
Not at all	217 (8.0)
A little	253 (8.0)
Somewhat	785 (23.9)
Well	1,036 (29.0)
Very well	1,149 (31.1)
**FCH communication with biological relatives** b	
Mother	1,706 (54.4)
Father	1,075 (35.2)
Sister(s)	1,042 (28.1)
Brother(s)	766 (21.0)
Children	723 (16.9)
Other biological family members	775 (24.0)
At least 1 biological family member	2,398 (70.0)
Multiple biological family members	1,876 (55.4)
None of the above	758 (24.2)
**FCH communication with a health care provider**	1,450 (39.0)
**Confidence completing FCH on medical forms**	
Not confident at all	398 (13.5)
A little confident	333 (11.4)
Somewhat confident	888 (25.5)
Very confident	943 (27.4)
Completely confident	860 (22.2)

a Missing data for variables included FCH knowledge (n = 64), FCH communication with biological relatives (n = 106), FCH communication with a health care provider (n = 106), and confidence completing FCH on medical forms (n = 82). Thus, categories may not add to 3,504.

b More than one response category could be selected.

Seventy percent of the population reported discussing FCH with at least 1 biological relative (Table 2). These discussions occurred most frequently with biological mothers (54.4%), followed by fathers (35.2%), sisters (28.1%), brothers (21.0%), and children (16.9%) (Table 2). One-quarter of the population also indicated that they had discussed FCH with other biological family members. More than half (55.4%) of the population had discussed this information with multiple biological family members.

Thirty-nine percent of the population discussed their FCH with a health care provider. Only half (49.6%) indicated that they were “very confident” or “completely confident” that they could complete their FCH on a medical form. Additionally, 25.5% indicated being “somewhat confident” and 24.9% indicated being “a little confident” or “not confident at all” in completing this task.

FCH knowledge differed by sex, annual household income, education, and race and ethnicity. FCH knowledge was positively correlated with being female (vs male), having an annual household income of $50,000 or more (vs <$20,000), and being a high school graduate (vs <high school graduate), and negatively correlated with being in the “other” racial/ethnic category (vs non-Hispanic White). Personal history of cancer was not significantly associated with FCH knowledge (Table 3).

**Table 3:** Multivariable Logistic Regression Models Predicting Each Family Cancer History (FCH) Variable, Health Information National Trends Survey (HINTS) 5, Cycle 2, 2018^a^

Characteristic	High Level of FCH Knowledge^b^	Discussed FCH With ≥1 Biological Relative^c^	Discussed FCH With a Health Care Provider^d^	High Level of Confidence Completing FCH on Medical Forms^e^
**Sex**				
Male	1 [Reference]	1 [Reference]	1 [Reference]	1 [Reference]
Female	2.26 (1.67–3.05)^f^	2.30 (1.70–3.11)^f^	2.20 (1.63–2.96)^f^	2.03 (1.52–2.73)^f^
**Age, y**				
18–34	1 [Reference]	1 [Reference]	1 [Reference]	1 [Reference]
35–49	1.21 (0.81–1.81)	0.68 (0.36–1.26)	1.75 (1.06–2.89)	1.27 (0.85–1.90)
50–64	1.39 (0.96–2.00)	0.55 (0.27–1.13)	1.50 (0.95–2.36)	1.40 (0.93–2.12)
65–74	1.30 (0.85–1.99)	0.56 (0.27–1.19)	1.38 (0.81–2.33)	1.29 (0.82–2.02)
≥75	1.69 (0.97–2.93)	0.30 (0.14–0.64)^f^	0.98 (0.48–2.01)	1.44 (0.82–2.54)
**Marital status**				
Not married or partnered	1 [Reference]	1 [Reference]	1 [Reference]	1 [Reference]
Married or partnered	0.87 (0.67–1.62)	0.93 (0.68–1.27)	1.01 (0.77–1.33)	1.03 (0.80–1.65)
**Annual household income, $**				
0–19,999	1 [Reference]	1 [Reference]	1 [Reference]	1 [Reference]
20,000–34,999	1.37 (0.77–2.44)	1.71 (0.90–3.25)	1.59 (0.88–2.90)	1.54 (0.90–2.63)
35,000–49,999	1.40 (0.88–2.21)	1.43 (0.83–2.44)	1.50 (0.85–2.64)	1.43 (0.87–2.35)
50,000–74,999	1.83 (1.01–3.33)^f^	2.54 (1.38–4.69)^f^	3.10 (1.75–5.49)^f^	1.43 (0.87–2.35)
≥75,000	2.21 (1.23–3.98)^f^	2.77 (1.65–4.63)^f^	2.73 (1.46–5.13)^f^	2.23 (1.37–2.63)^f^
**Education**				
<High school graduate	1 [Reference]	1 [Reference]	1 [Reference]	1 [Reference]
High school graduate	1.63 (0.89–2.96)	1.34 (0.66–2.72)	1.10 (0.41–2.96)	1.82 (0.99–3.36)
Technical or vocational school or some college	1.95 (1.07–3.56)^f^	2.64 (1.38–5.03)^f^	1.98 (0.78–5.05)	2.46 (1.38–4.39)^f^
≥College graduate	2.29 (1.30–4.01)^f^	2.47 (1.27–4.80)^f^	2.46 (1.06–5.71)^f^	2.82 (1.67–4.76)^f^
**Race/ethnicity**				
Non-Hispanic White	1 [Reference]	1 [Reference]	1 [Reference]	1 [Reference]
Non-Hispanic Black	0.73 (0.45–1.19)	0.43 (0.24–0.75)^f^	0.48 (0.32–0.72)^f^	0.89 (0.53–1.50)
Hispanic	0.78 (0.50–1.21)	0.48 (0.30–0.79)^f^	0.57 (0.38–0.85)^f^	0.87 (0.56–1.36)
Other	0.47 (0.29–0.77)^f^	0.47 (0.21–1.06)	0.63 (0.34–1.17)	0.59 (0.33–1.07)
**Metropolitan area**				
Urban	1 [Reference]	1 [Reference]	1 [Reference]	1 [Reference]
Rural	1.30 (0.88–1.91)	0.79 (0.50–1.26)	1.34 (0.83–2.18)	1.15 (0.80–1.65)
**Personal cancer history**				
No	1 [Reference]	1 [Reference]	1 [Reference]	1 [Reference]
Yes	1.04 (0.67–1.62)	1.90 (1.24–2.92)^f^	3.02 (1.81–5.05)^f^	1.30 (0.86–1.95)

a All values are odds ratio (95% CI).

b Reference is low level of FCH knowledge. Survey responses to question on how well respondent knows FCH were dichotomized into a high level of FCH knowledge (very well or well) and a low level FCH knowledge (somewhat, a little, or not at all).

c Reference group is no discussion.

d Reference group is no discussion.

e Reference is low level of confidence. Survey responses to question on how confident respondent is about completing a summary of FCH on a medical form were dichotomized into high level of confidence (completely confident or very confident) and low level of confidence (somewhat, a little, or not at all confident).

f Significant at *P* < .05.

FCH communication with at least 1 biological relative also differed by demographic characteristics, specifically sex, age, annual household income, education, race and ethnicity, and personal cancer history. Women (vs men), people with an annual household income of $50,000 or more (vs <$20,000), people who attended technical or vocational school or some college and college graduates (vs <high school graduate), and people with a personal cancer history (vs no personal cancer history) were more likely to report having had a discussion with at least 1 biological relative. People aged 75 or older (vs 18–34), and non-Hispanic Black and Hispanic (vs non-Hispanic White) people were less likely to report having had a discussion about FCH with at least 1 biological relative (Table 3).

Women (vs men), people with at least $50,000 (vs <$20,000) in annual household income, and college graduates (vs <high school) were more likely to have had an FCH discussion with a health care provider. People with a personal history of cancer were also more likely than people without such a history to report having had a discussion. However, non-Hispanic Black and Hispanic people were less likely than non-Hispanic White people to have had a discussion with a health care provider (Table 3).

Only sex, annual household income, and education were correlated with confidence completing FCH on medical forms. A high level of confidence in the ability to complete this task was associated with being female, having the highest annual household income level (≥$75,000 vs < $20,000), and having at least attended technical or vocational school or some college (vs <high school graduate). Personal cancer history was not associated with confidence completing FCH on medical forms (Table 3).

## Discussion

FCH plays a role in individual cancer risk; thus, it is important for people to communicate this history with family members and health care providers. Our study identified demographic correlates of FCH knowledge, FCH communication with biological relatives and health care providers, and confidence completing FCH on medical forms among the US adult population. Results can inform future research targeting or tailoring FCH communication interventions for adult populations. Additionally, results offer several practical insights that can be used to promote communication about FCH among families and health care providers.

A precise family history documenting cancers among biological relatives “is the basis for state-of-the-art cancer risk assessment” (16). Despite agreement that knowledge of one’s family health history is important (17), our study found that less than one-third of US adults know their family’s cancer history very well and one-quarter have not discussed FCH with any biological relatives. These gaps in FCH knowledge and communication may negatively affect the ability to accurately evaluate inherited cancer risks across a substantive portion of the population.

Results also shed light on demographic differences and potential disparities in FCH knowledge and communication. Building on studies from smaller, community-based settings (7,11,13), our findings highlighted sex differences in several aspects of FCH communication. Consistent with previous research (11,13,14), women were more likely than men to report FCH knowledge and communication with relatives and health care providers. Additionally, mothers were the most frequent biological relative with whom study respondents discussed FCH. This finding is not surprising because women are often the keepers of family health information (14). Women’s role as disseminators of FCH information may also help explain the finding that women were more likely than men to report high levels of confidence in documenting their family cancer histories on medical forms. Future interventions could consider how to more effectively engage men in discussions about FCH.

FCH communication with relatives also differed by race and ethnicity. Non-Hispanic Black and Hispanic people were less likely than non-Hispanic White people to communicate about FCH with biological relatives, consistent with previous research (7,18,19). For example, Corona et al found that less than one-fifth of Latino young adults had constructed an FCH or shared hereditary cancer risk information with relatives (18). Health beliefs and perceptions may account for some observed differences in FCH communication among non-White populations. Several studies have identified intergenerational tension or hesitation to share health information, which is perceived to be private, as a barrier to FCH communication among older and younger relatives (7,12). Recently, Hood drew attention to the importance of incorporating cultural context and addressing racial/ethnic disparities in family health history research and development of culturally appropriate interventions to increase family communication about health (20). One promising example, a Spanish-language digital family health history tool, demonstrated acceptability and usability among Spanish-speaking families for collecting health information among a culturally and linguistically diverse population (21).

FCH communication with biological relatives also differed by socioeconomic status and age group. People with low annual household income and educational attainment were less likely to report FCH communication than their counterparts with more income and education. Because such people may be less likely to have access to medical care, including genetic testing, FCHs may be important communication tools for decreasing disparities in the reach of personalized medicine (22). Interestingly, people aged 75 or older were less likely than people aged 18 to 34 to report discussing FCH with at least 1 biological family member, even though they may be more likely to accumulate knowledge of diagnoses across family generations.

Finally, more than 60% of US adults had not discussed FCH with a health care provider. Our results highlighted differences in FCH discussions with health care providers by sex, annual household income, education, race and ethnicity, and personal cancer history, suggesting population subgroups that may benefit from targeted and tailored efforts to address common barriers and promote FCH discussions. Previous research noted numerous barriers to patient–provider discussions about family histories, including lack of time during medical appointments, limited knowledge of FCH reported by patients, and lack of systematic collection and interpretation of family history information (16,23). Additionally, complex underlying factors, such as medical mistrust and patient concerns about harmful use of genetic information, present system-level barriers to patient–provider communication about FCH among non-White populations (24).

Despite these barriers, opportunities exist for improving patient–provider communication about FCH and supporting these discussions at the point of care (25,26). These opportunities include training on communication skills for health care providers and system-level interventions that include standardization of key elements of a precise family history (eg, cancer types, age at onset), consistent information structures for health history intake, and integration of family health history data into patients’ electronic health records for clinical decision support (26).

Although analyzing a nationally representative sample of the US adult population strengthened our findings, our study also had several limitations. It was limited by reliance on self-reported and cross-sectional data; thus, causal relationships between variables cannot be inferred. Future research could address this limitation by using longitudinal survey methodology. Additionally, because of the limitations of the data set, we could not discern the direction, frequency, duration, depth, or timing of discussions with relatives or health care providers or reasons why individuals may not engage in FCH discussions. Qualitative studies complement these population-level findings to better understand the complex picture of barriers to and facilitators of FCH communication (7,12,24). For example, a recent systematic review identified 13 qualitative and mixed-methods studies examining family risk communication among young adults with a positive *BRCA1/2* test result and highlighted future directions for genetic counseling and educational interventions (27). Additionally, various definitions of “family” that include biological and social relationships affect observed differences in FCH communication (6,8). Thus, a potential limitation of this study is that the HINTS survey did not capture diverse conceptualizations of “family” that may include nonbiological relatives who nonetheless engage in FCH discussions (eg, spouses, in-laws, godparents).

Documented family cancer histories can be used to identify people at increased risk of cancer and help personalize plans for cancer prevention and early detection. For example, a recent study found family history criteria helped identify high-risk patients who would benefit from early colorectal cancer screening (28). To this end, digital tools, such as the US Surgeon General’s My Family Health Portrait, could be promoted to support tracking of family health history (29–31). However, effective use of family cancer histories will entail supporting communication among individuals, families, and health care providers as well as health care systems–level support for systematic FCH collection and clinical decision making (8,17,26). Continued surveillance of FCH communication behavior is recommended as personalized medicine advances and access to genetic testing expands among the population.
